# Supplementary material for: Empirical methods for controlling false positives and estimating confidence in ChIP-Seq peaks
Source: BMC Bioinformatics. 2008 Dec 5;9:523. doi: 10.1186/1471-2105-9-523 (PMC2628906; doi:10.1186/1471-2105-9-523)
Supplement: Additional File 1 — A variety of html documents from the USeq web site detailing the available applications, their best usage, output file type descriptions, command line menus, etc. [file 1471-2105-9-523-S1.zip › USeqUserGuides/JavaDoc.zip!/JavaDoc/gata/main/class-use/GATAParams.html]

Uses of Class gata.main.GATAParams


|  |  |  |
| --- | --- | --- |
| **Overview** \| **Package** \| **Class** \| **Use** \| **Tree** \| **Deprecated** \| **Index** \| **Help** | |  |
| PREV   NEXT | **FRAMES**    **NO FRAMES**     **All Classes** |  |


---


## **Uses of Class gata.main.GATAParams**

| Packages that use GATAParams | |
| --- | --- |
| **gata.geneGlyphs** | For representing annotation, scale bars, and generic tracks. |
| **gata.main** | Main app, utility, and params for GATAPlotter. |
| **gata.menu** | GATAPlotter menus, panels, and frames. |
| **gata.plotter** | GATAPlotter primary panel, sliders, tools, and conservation panels. |

Uses of GATAParams in gata.geneGlyphs

Constructors in gata.geneGlyphs with parameters of type GATAParams

`Annotation(AnnoSpecParams annoParams,
GATAParams gataParams)`

`GeneRepGlyph(GeneRep geneRep,
GATAParams gataParams,
AnnoSpecParams annoParams)`

`GlyphPanel(AnnoSpecParams annoParams,
GATAParams gataParams)`

`ScaleBar(AnnoSpecParams annoParams,
GATAParams gataParams)`

`TransGrpGlyph(TransGroup transGrp,
GATAParams gataParams,
AnnoSpecParams annoParams)`

Uses of GATAParams in gata.main

| Methods in gata.main with parameters of type GATAParams | |
| --- | --- |
| `void` | `PlotterPreferences.fetchAndAssignGATAParams(GATAParams p)` |
| `void` | `PlotterPreferences.setCurrentPlotterPreferences(GATAParams p)`             Contains all the default preferences for GATAParams |
| `void` | `PlotterPreferences.setPlotterDefaults(GATAParams p)`             Contains all the default preferences for GATAParams, alter these to change the default view |

Uses of GATAParams in gata.menu

Constructors in gata.menu with parameters of type GATAParams

`FileInputForm(GATAParams params)`

`FileInputPanel(GATAParams params,
javax.swing.JFrame frame)`

`GATAFrame(GATAParams gataParams)`

`SaveImageForm(GATAParams params)`

`SaveImagePanel(GATAParams params,
javax.swing.JFrame frame)`

Uses of GATAParams in gata.plotter

Constructors in gata.plotter with parameters of type GATAParams

`AlignPanel(GATAParams params)`

`ConservedSeqs(GATAParams params)`

`Sliders(AlignParams ap,
GATAParams params)`

`ToolsFrame(int locX,
int locY,
GATAParams params)`

---


|  |  |  |
| --- | --- | --- |
| **Overview** \| **Package** \| **Class** \| **Use** \| **Tree** \| **Deprecated** \| **Index** \| **Help** | |  |
| PREV   NEXT | **FRAMES**    **NO FRAMES**     **All Classes** |  |


---
